# Regulation of nerve-evoked contractions of the murine vas deferens

**DOI:** 10.1007/s11302-024-09993-y

**Published:** 2024-02-20

**Authors:** Pei Yee Wong, Zhihui Fong, Mark A. Hollywood, Keith D. Thornbury, Gerard P. Sergeant

**Affiliations:** 1 Smooth Muscle Research Centre, Dundalk Institute of Technology, Dublin Road, Dundalk, Co. Louth Ireland; 2 Department of Physiology and Membrane Biology, School of Medicine, University of California, Davis, CA 95616 USA

**Keywords:** Vas deferens, Smooth muscle, PKC, Contraction, ATP, Synergism

## Abstract

Stimulation of sympathetic nerves in the vas deferens yields biphasic contractions consisting of a rapid transient component resulting from activation of P2X1 receptors by ATP and a secondary sustained component mediated by activation of α_1_-adrenoceptors by noradrenaline. Noradrenaline can also potentiate the ATP-dependent contractions of the vas deferens, but the mechanisms underlying this effect are unclear. The purpose of the present study was to investigate the mechanisms underlying potentiation of transient contractions of the vas deferens induced by activation of α_1_-adrenoceptors. Contractions of the mouse vas deferens were induced by electric field stimulation (EFS). Delivery of brief (1s duration) pulses (4 Hz) yielded transient contractions that were inhibited tetrodotoxin (100 nM) and guanethidine (10 µM). α,β-meATP (10 µM), a P2X1R desensitising agent, reduced the amplitude of these responses by 65% and prazosin (100 nM), an α_1_-adrenoceptor antagonist, decreased mean contraction amplitude by 69%. Stimulation of α_1_-adrenoceptors with phenylephrine (3 µM) enhanced EFS and ATP-induced contractions and these effects were mimicked by the phorbol ester PDBu (1 µM), which activates PKC. The PKC inhibitor GF109203X (1 µM) prevented the stimulatory effects of PDBu on ATP-induced contractions of the vas deferens but only reduced the stimulatory effects of phenylephrine by 40%. PDBu increased the amplitude of ATP-induced currents recorded from freshly isolated vas deferens myocytes and HEK-293 cells expressing human P2X1Rs by 93%. This study indicates that: (1) potentiation of ATP-evoked contractions of the mouse vas deferens by α_1_-adrenoceptor activation were not fully blocked by the PKC inhibitor GF109203X and (2) that the stimulatory effect of PKC on ATP-induced contractions of the vas deferens is associated with enhanced P2X1R currents in vas deferens myocytes.

**Supplementary Information:**

The online version contains supplementary material available at 10.1007/s11302-024-09993-y.

## Introduction

The vas deferens is a muscular tube which acts as a conduit for transport of sperm from the epididymis to the urethra during ejaculation [1, 2]. Propulsion of sperm is achieved by contractions of smooth muscle in the wall of the vas deferens which are induced by co-release of ATP and noradrenaline from sympathetic nerves [3–6]. In most species stimulation of nerves in the vas deferens yields a biphasic response, consisting of a rapid transient component, sometimes referred to as a ‘twitch’ contraction, and a secondary sustained component [2, 7, 8]. Studies on guinea-pig and rabbit vas deferens [9–12], demonstrated that blockade of P2X1Rs abolished the initial transient component of the response without affecting the second component, whereas inhibition of α_1_-ARs inhibited the second component of the response without affecting the transient phase. Therefore, it is widely thought that the transient component of nerve-evoked contractions of the vas deferens is exclusively mediated by activation of P2X1Rs, whereas the sustained component only involves activation of α_1_-ARs. However, studies on rat vas deferens showed that inhibition of α_1_-ARs reduced the amplitude of both the transient and sustained components of EFS-evoked contractions [13]. It is now recognised that noradrenaline can augment ATP-induced contractions of the vas deferens [14–16] and that the inhibitory effects of prazosin on the transient component result from inhibition of the facilitatory effects of α_1_-AR activation on contractions induced by ATP. Therefore, there is greater complexity in the mechanisms underlying nerve-evoked contractions of the vas deferens than is widely acknowledged.

White et al., (2013) showed that dual genetic deletion of α_1_-ARs and P2X1Rs in male mice resulted in 100% infertility, without effects on sexual behaviour [17]. Therefore, it is crucial to gain a better understanding of the mechanisms that underpin synergism between the effects of ATP and noradrenaline on contractions of the vas deferens as this is likely to affect male fertility. Smith & Burnstock, (2004) demonstrated that activation of PKC in guinea-pig vas deferens potentiated responses to ATP and that inhibition of PKC attenuated the stimulatory effects of noradrenaline on subsequent responses to ATP, suggesting a role for PKC in the stimulatory effects of noradrenaline on ATP responses [14]. However, Fujita et al., (1995) found that although activation of PKC with the phorbol ester PDBu mimicked the effects of noradrenaline, the effects of noradrenaline were not affected by PKC(19–31), a PKC inhibitor peptide [15]. Therefore, while it is evident that PKC can potentiate ATP responses in the vas deferens it not clear if the stimulatory effects of noradrenaline on ATP responses are mediated by PKC. The purpose of the present study was to examine the contribution of PKC to the stimulatory effects of α_1_-AR activation on ATP responses in mouse vas deferens and the mechanisms which may underlie these effects.

## Materials & methods

### Tissue dissection

All procedures were carried out in accordance with current EU legislation and with the approval of Dundalk Institute of Technology Animal Ethics Committee. Male C57BL/6 wild-type (WT) mice aged 10–16 wks old were humanely killed by intraperitoneal injection of pentobarbitone (100 mg/kg). Vasa deferentia were removed and placed in Krebs’ solution. Each vas deferens was pinned to a Sylgard-coated dissection dish containing Krebs’ solution. Adherent fat, blood vessels and connective tissue were carefully removed by sharp dissection under a dissecting microscope. A small syringe with a small-gauge needle was carefully inserted into the prostatic end of the vas deferens to flush out semen. The vas deferens was cut into epididymal and prostatic segments, approximately 10–15 mm in length for isometric tension recording or transferred to Ca^2+^-free Hanks’ solution for enzymatic digestion.

### Vas deferens smooth muscle cell isolation

Vas deferens segments were cut into 1 mm^3^ pieces and stored in Ca^2+^-free Hanks’ solution for 15 min at 4˚C prior to cell dispersal. Tissue pieces were incubated in dispersal medium containing (per 5 ml) of Ca^2+^-free Hanks’ solution: 10 mg collagenase (Sigma type 1 A), 1 mg proteinase (Sigma type XXIV), 10 mg bovine serum albumin (Sigma) and 10 mg trypsin inhibitor (Sigma) for 7–8 min at 37˚C. Tissue was then transferred to Ca^2+^-free Hanks’ solution and stirred for a further 7–8 min to release single smooth muscle cells. These were plated in Petri dishes containing 100 µM Ca^2+^ Hanks’ solution and allowed to settle in glass bottomed Petri dishes until they had stuck down.

### Isometric tension recordings

Longitudinal segments of vas deferens were mounted in water-jacketed organ baths, perfused with warmed Krebs’ solution, adjusted to 5 mN tension, and equilibrated for 40 min. Isometric contractions were recorded using a Myobath system, and data acquired using DataTrax2 software (WPI). Electrical field stimulation (EFS) was used to excite transmural nerves and was applied via two platinum electrode wires (5 mm length, 2.5 mm apart) by a MultiStim system-D330 stimulator (Digitimer Ltd, England). Two different EFS protocols were used in this study. The first examined the effect of EFS at a single frequency (4 Hz), applied for a duration of 1 s at 100 s intervals and the second used a range of frequencies (1, 2, 4, 8 and 16 Hz) for durations of 30 s at 20 min intervals. Mean contraction amplitude of contractions evoked by the former protocol was obtained by averaging peak contraction amplitude of ten EFS-induced contractions before and during drug-addition (when they had their maximal effect). Drugs were added directly to the organ bath, where they were diluted in Krebs’ solution to their final concentration. GF109203X is regarded as a potent inhibitor of PKC with IC_50_ values in the nanomolar range [18]. However, at concentrations above 1 µM GF109203X, and other structurally-related PKC inhibitors, have been reported to have non-selective effects including inhibition of nicotinic and muscarinic acetylcholine receptors [19, 20]) and voltage-dependent Na^+^ channels [21]. We opted to use GF109203X at a concentration of 1 µM to maximise its inhibitory effects on PKC while minimising the risk of non-selective effects associated with higher concentrations.

### Electrophysiology

The perforated patch configuration of the whole cell patch clamp technique was used to record ATP-induced currents from freshly isolated vas deferens smooth muscle cells (VDSMC) or HEK-293 cells transiently transfected with human P2X1 (NM_002558, Origene Technologies) plasmid construct (200 ng ml^− 1^) using Lipofectamine 2000 (Invitrogen). Electrical access between the pipette and cell interior was achieved by inclusion of the pore forming compound amphotericin B (420 µg/mL) in the pipette solution. Voltage clamp commands were delivered via an Axopatch 1D patch clamp amplifier (Molecular Devices, Sunnyvale, CA, USA) connected to a Digidata 1440 A Digitizer (Axon Instruments) interfaced to a computer running pClamp software (Axon Instruments). During experiments, the dish containing the cells was superfused with Hanks’ solution. In addition, the cell under study was continuously superfused by means of a close delivery system consisting of a pipette (tip diameter 200 μm) placed approximately 200 μm away. This could be switched, with a dead-space time of < 5 s, to a solution containing a drug. Cells were held at -60 mV and ATP (1 µM) was applied for five second durations. P2X1 currents were reproducible at four and seven-minute intervals for VDSMC and HEK-293 cells, respectively, and two reproducible responses to ATP were obtained under control conditions in each experiment prior to addition of drugs.

### Drugs

α,β-meATP (10 µM, Tocris), prazosin hydrochloride (100 nM, Abcam), Phorbol 12,13-dibutyrate (PDBu, 1 µM Sigma-Aldrich), GF109203X (1 µM Merck), ATP (1 µM, Sigma-Aldrich), phenylephrine (3 µM Sigma-Aldrich).

### Solutions

Solutions used were of the following composition (mM): *Krebs’ solution*: 120 NaCl, 5.9 KCl, 25 NaHCO_3_, 1.2 NaH_2_PO_4_·2H_2_O, 5.5 glucose, 1.2 MgCl_2_, and 2.5 CaCl_2_. pH was adjusted to 7.4 by bubbling the solution with 95% O_2_–5% CO_2_. *Hanks’ Solution*: NaCl (125.0), KCl (5.4), Glucose (10.0), Sucrose (2.9), NaHCO_3_ (4.2), KH_2_PO_4_ (0.4), NaH_2_PO_4_ (0.3), MgCl_2_.6H_2_O (0.5), CaCl_2_.2H_2_O (1.8), MgSO_4_ (0.4), HEPES (10.0). pH to 7.4 using NaOH. *Ca*^*2+*^*-free Hanks’ Solution*: NaCl (125), KCl (5.36), Glucose (10), Sucrose (2.9), NaHCO_3_ (15.5), Na_2_HPO_4_ (0.33), KH_2_PO_4_ (0.44), HEPES-free acid (10). pH to 7.4 with NaOH. *Perforated patch pipette solution*: CsCl (133), MgCl_2_ (1.0), EGTA (0.5), HEPES (10), pH adjusted to 7.2 with CsOH.

### Data analysis and statistics

Experimental series were obtained from three or more animals; *n* refers to the number of tissue segments or cells studied and *N* to the number of animals. Data were analysed using Prism software (GraphPad). Summary data are presented as mean ± SEM. Statistical comparisons were performed on original (non-normalised) data using either Student’s paired *t*-test or, if three experimental groups were compared, ANOVA followed by Tukeys’ *post hoc* test, with *p* < 0.05 considered statistically significant.

## Results

**Fig. 1:**
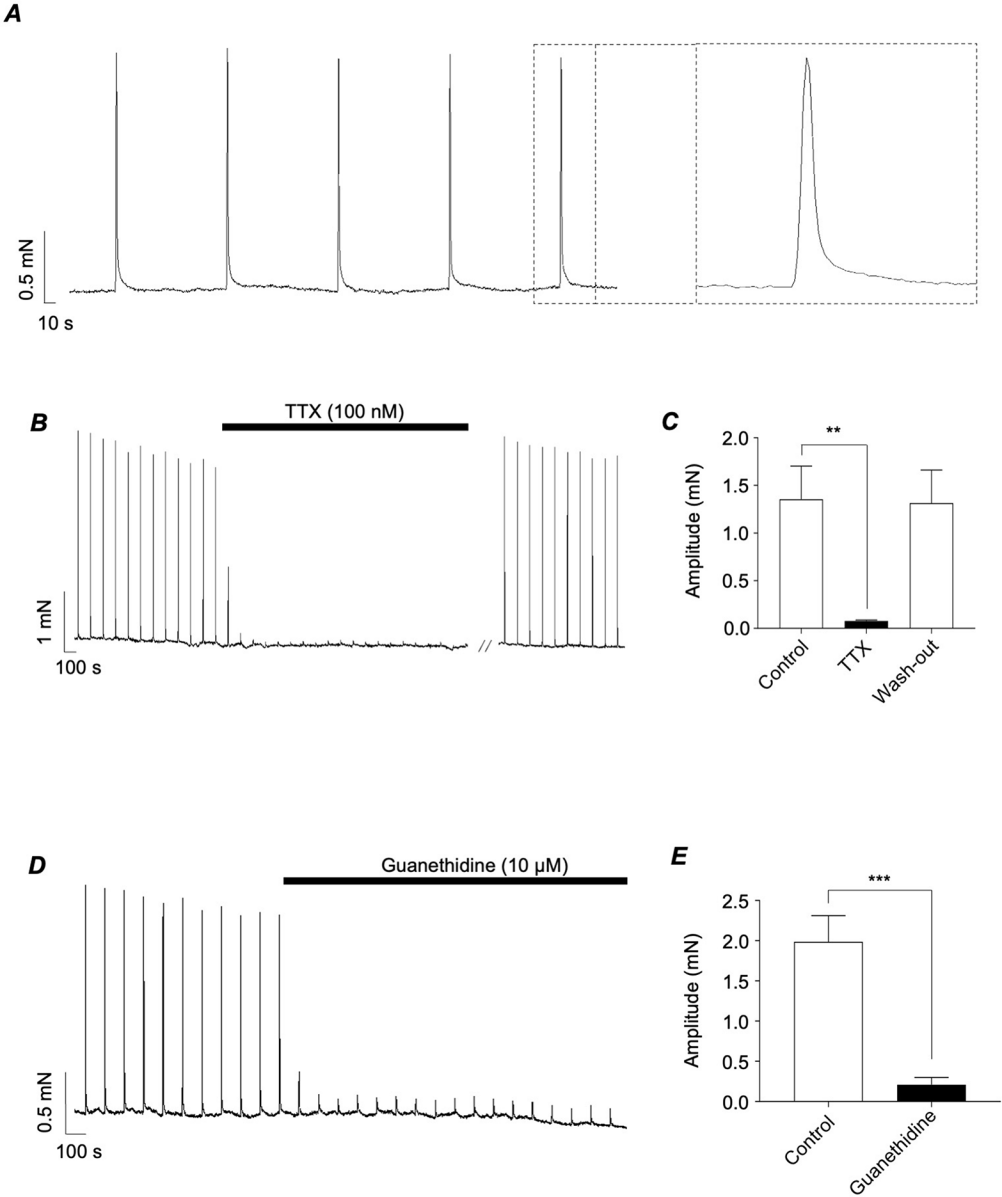
*A*, representative tension recording from murine vas deferens showing contractions induced by EFS (4 Hz, 1 s duration). B, representative trace showing effects of TTX (100 nM). *C*, summary bar chart plotting mean amplitude of EFS-evoked contractions before, during and following wash-out of TTX. *D*, representative tension recording from murine vas deferens showing contractions induced by EFS (4 Hz, 1 s duration) before and during the presence of guanethidine (10 µM).E*D*, summary bar chart plotting mean amplitude of EFS-evoked contractions before and during the presence of guanethidine. Error bars represent SEM. **p < 0.01; ***p < 0.001

Experiments were performed to examine the mechanisms underlying transient EFS-evoked contractions of the mouse vas deferens. The EFS protocol used for this series of experiments involved delivery of brief (1 s duration) pulses at 100 s intervals at a frequency of 4 Hz. This yielded reproducible monophasic contractions (Fig. 1A) that were inhibited by TTX (100 nM, Fig. 1B&C) and guanethidine (10 µM, Fig. 1D&E), which prevents release of neurotransmitters from sympathetic nerves [22].

**Fig. 2:**
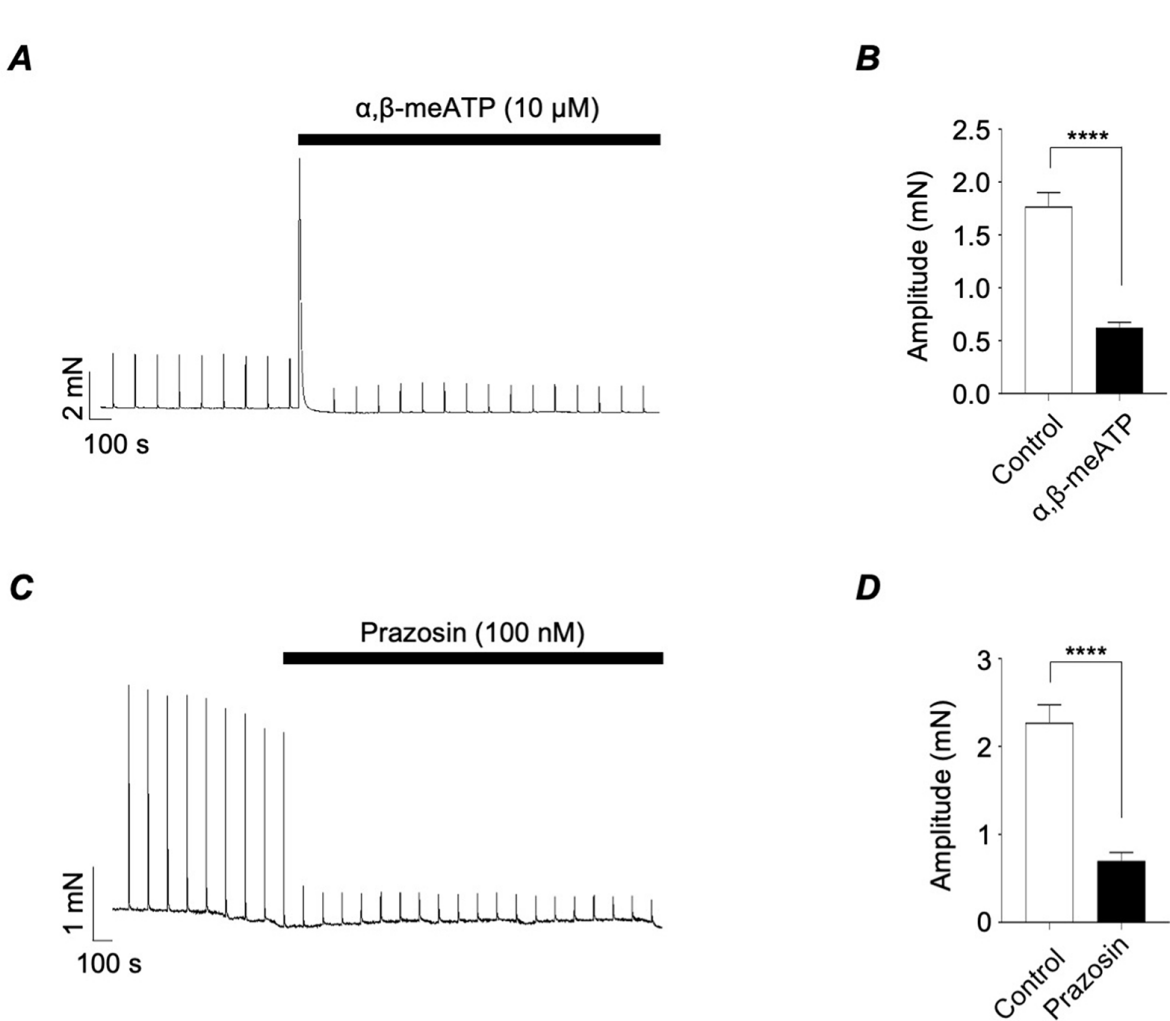
*A*, representative tension recording from murine vas deferens showing contractions induced by EFS (4 Hz, 1 s duration) before and during the presence of α,β-meATP (10 µM). *B*, summary bar chart plotting mean amplitude of EFS-evoked contractions before, during and following wash-out of α,β-meATP. *C*, representative tension recording from murine vas deferens showing contractions induced by EFS (4 Hz, 1 s duration) before and during the presence of prazosin (100 nM). *D*, summary bar chart plotting mean amplitude of EFS-evoked contractions before and during the presence of prazosin. Error bars represent SEM. **p < 0.01; ***p < 0.001

To assess the contribution of α_1_-ARs and P2X1Rs to these contractions we examined the effects of prazosin, an α_1_-AR antagonist and α,β-meATP, a desensitising agonist of P2X1Rs. Figure 2A is a representative trace showing that application of α,β-meATP (10 µM) induced a large transient contraction and then reduced the amplitude of the EFS responses. The summary bar chart in Fig. 2B shows that α,β-meATP reduced mean contraction amplitude by 65% (*p* < 0.0001; *N* = 34; *n* = 20). Prazosin (100 nM) also inhibited EFS-evoked contractions (Fig. 2C), reducing mean contraction amplitude by 69% (Fig. 2D, *p* < 0.0001; *N* = 34; *n* = 20).

Data shown in supplementary Fig. 1 confirm that EFS for longer durations (30 s) induced frequency-dependent biphasic contractions of the vas deferens that were comprised of an initial transient component that peaked within 2 s and a sustained component that was maintained with the stimulus duration. Prazosin (100 nM) reduced the amplitude of both the transient and sustained phases of the response. Subsequent addition of α,β-meATP (10 µM) further attenuated the remaining responses (Supplementary Fig. 1A-C). Similarly, when α,β-meATP was added first, before addition of prazosin, it reduced the amplitude of the transient and sustained components of the response.

**Fig. 3:**
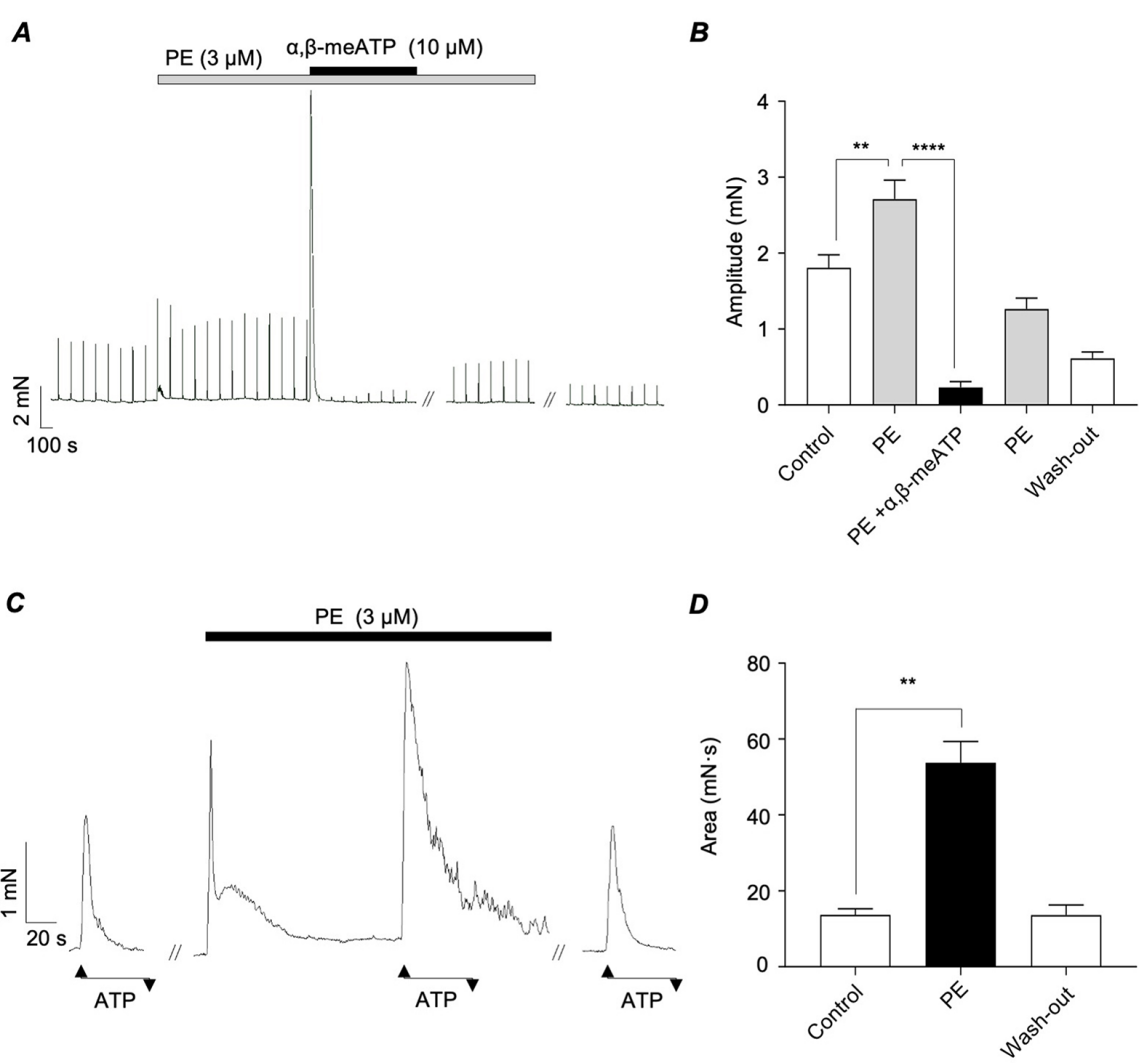
*A*, Representative tension recording from murine vas deferens showing that EFS-evoked contractions were enhanced by phenylephrine (PE, 3 µM) and that these responses were abolished by α,β-meATP (10 µM). *B*, summary bar chart plotting mean amplitude of EFS responses before, during PE, PE + α,β-meATP and wash-out. *C*, representative trace showing effects of PE on contractions induced by ATP (1 µM). *D*, summary bar chart plotting mean area of ATP-induced contractions before, during and following wash-out of PE. Error bars represent SEM. **p < 0.01; ****p < 0.0001

Several studies have shown that purinergic nerve-evoked contractions of the vas deferens are potentiated by stimulation of α_1_-ARs and therefore the inhibitory effects of prazosin on transient ‘twitch’ contractions of the vas deferens may result from inhibition of the stimulatory effects of noradrenaline on contractions initiated by activation of postjunctional P2X1Rs. We investigated the effects of α_1_-AR activation on purinergic responses in the vas deferens using phenylephrine (PE), a α_1_-AR agonist. The representative trace in Fig. 3A shows that application of PE (3 µM) increased the amplitude of EFS-evoked contractions of the vas deferens and that these responses were reversibly inhibited by α,β-meATP (10 µM). PE increased mean contraction amplitude from 1.8 ± 0.2 to 2.7 ± 0.3 mN (*p* < 0.01 *N* = 4; *n* = 7) and addition of α,β-meATP reduced the responses to 0.2 ± 0.1 mN (*p* < 0.0001; *N* = 4; *n* = 7). PE increased the area of contractions induced by exogenous application of ATP by 293% from 13.7 ± 1.6 to 53.7 ± 5.6 mN·s (*p* < 0.01; *N* = 4; *n* = 6; Fig. 3C&D).

**Fig. 4:**
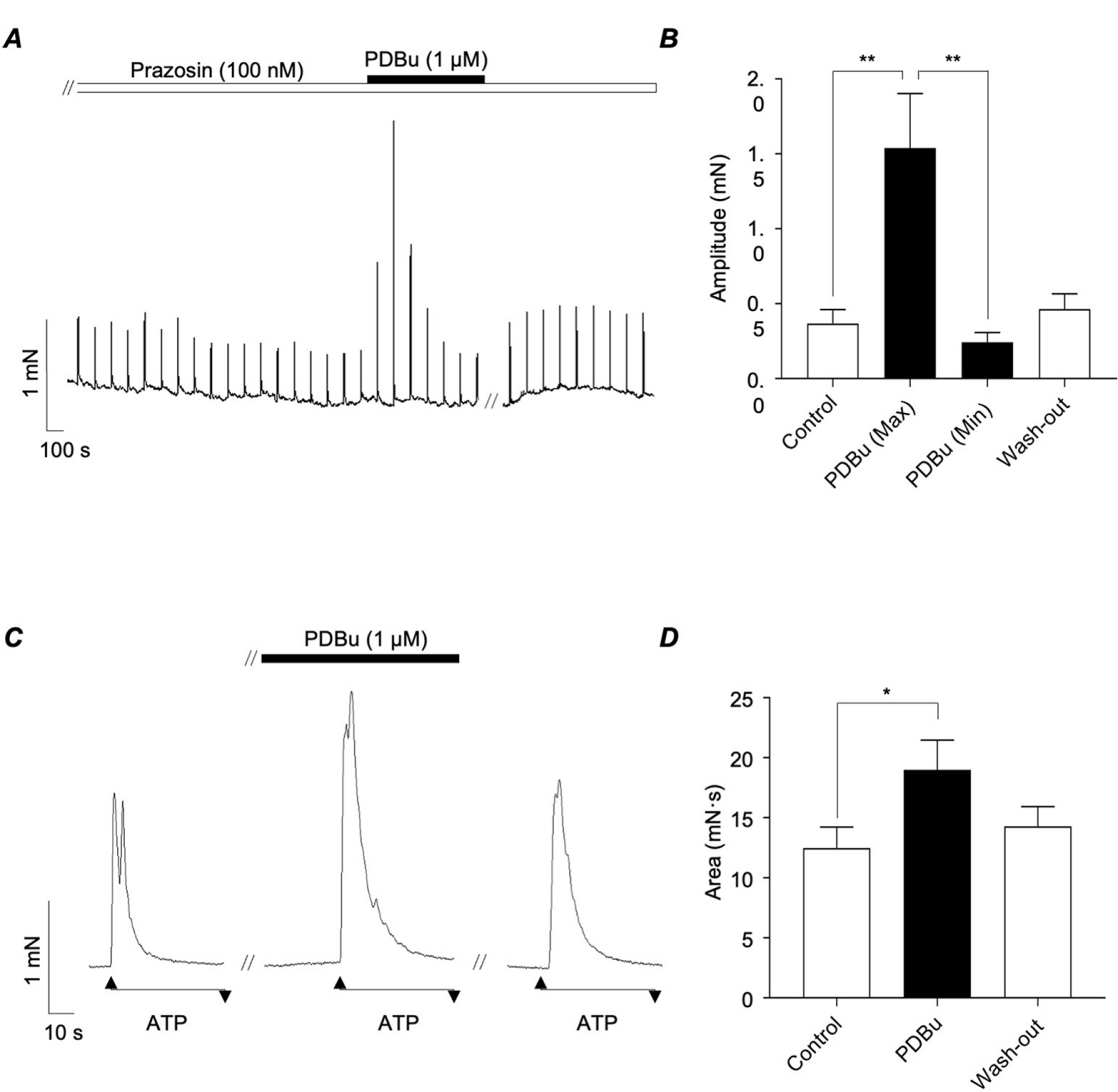
*A*, Representative tension recording from murine vas deferens showing that EFS-evoked contractions were transiently enhanced by the phorbol ester, PDBu (1 µM). These experiments were performed in the presence of prazosin (100 nM). *B*, summary bar chart plotting mean amplitude of EFS responses before, during PDBu (after 2 & 15 min incubation) and wash-out. *C*, representative trace showing effects of PDBu on contractions induced by ATP (1 µM). *D*, summary bar chart plotting mean amplitude of ATP-induced contractions before, during and following wash-out of PDBu. Error bars represent SEM. *p < 0.05; **p < 0.01

Smith & Burnstock, (2004) indicated that activation of PKC played a role in the potentiation of ATP-induced contractions of guinea-pig vas deferens by noradrenaline [14]. We tested if PKC activation enhanced the amplitude of purinergic responses in mouse vas deferens by examining the effects of PDBu, a recognised PKC activator [23]. These experiments were performed in the presence of prazosin to remove responses induced by activation of α_1_-ARs. The representative trace in Fig. 4A confirmed that application of PDBu (1 µM) transiently increased the amplitude of EFS-evoked contractions. In 8 preparations PDBu increased the mean amplitude of EFS responses by 320%, from 0.4 ± 0.1 to 1.5 ± 0.5 mN (*p* < 0.01; *N* = 8; *n* = 5). However, contraction amplitude decreased to 0.2 ± 0.1 mN after 15 min in the continued presence of PDBu (Fig. 4B). PDBu also enhanced the amplitude of responses induced by ATP (1 µM, Fig. 4C). Mean contraction area of ATP responses increased by 52% from 12.5 ± 1.7 to 19.0 ± 2.4 mN·s (Fig. 4D, *p* < 0.05; *N* = 5; *n* = 10).

**Fig. 5:**
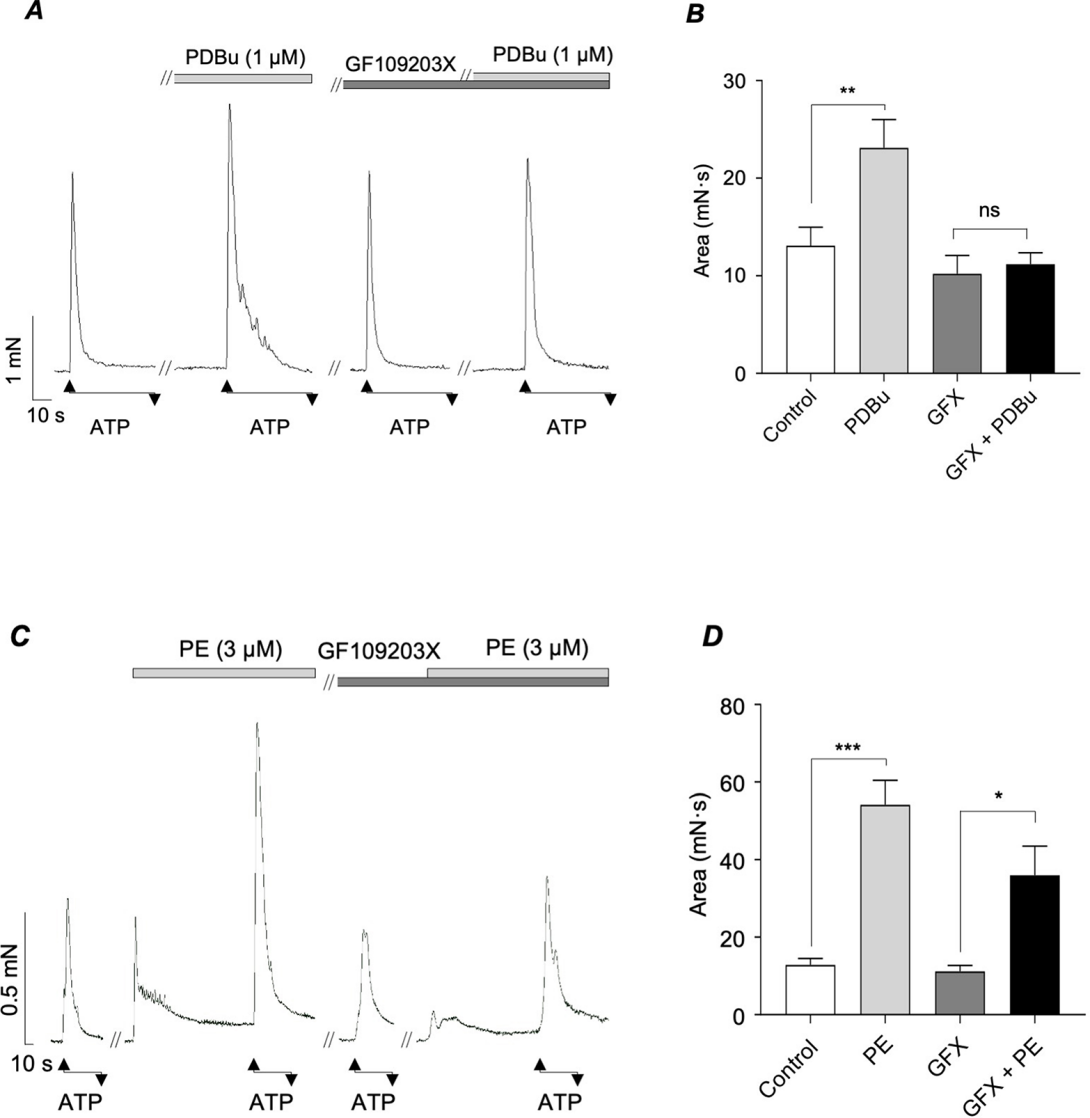
*A&B*, representative trace and summary bar chart showing the effects of PE (3 µM) on contractions induced by ATP under control conditions and in the presence of GF109203X (1 µM). *C&D*, representative trace and summary bar chart showing the effects of PDBu (1 µM) on contractions induced by ATP under control conditions and in the presence of GF109203X (1 µM). Error bars represent SEM. *p < 0.05; **p < 0.01; ***p < 0.001

To assess if PKC contributed to the stimulatory effects of PE, we examined the effects of PE on ATP-induced contractions before and during the presence of the PKC inhibitor GF109203X (1 µM). We first performed control experiments to confirm that GF109203X could inhibit PKC-dependent responses in the mouse vas deferens by testing its effects on ATP responses that were augmented by the PKC activator PDBu (1 µM). The results shown in Fig. 5A&B demonstrate that GF109203X inhibited the effects of PDBu on ATP responses, confirming that it was an effective inhibitor of PKC. The representative trace in Fig. 5C illustrates that PE was still capable of enhancing ATP-induced contractions in the presence of GF109203X. Overall, the summary data in Fig. 5D show that PE enhanced the mean area of ATP responses by 222% in the presence of GF109203X (*p* < 0.05; *N* = 6; *n* = 8) compared to 323% under control conditions (*p* < 0.001; *N* = 6; *n* = 8). Therefore, inhibition of PKC only reduced the stimulatory effects of PE on ATP responses by 40%. It should also be noted that ATP-evoked contractions were slightly reduced in the presence of GF109203X, but that this was not statistically significant.

**Fig. 6:**
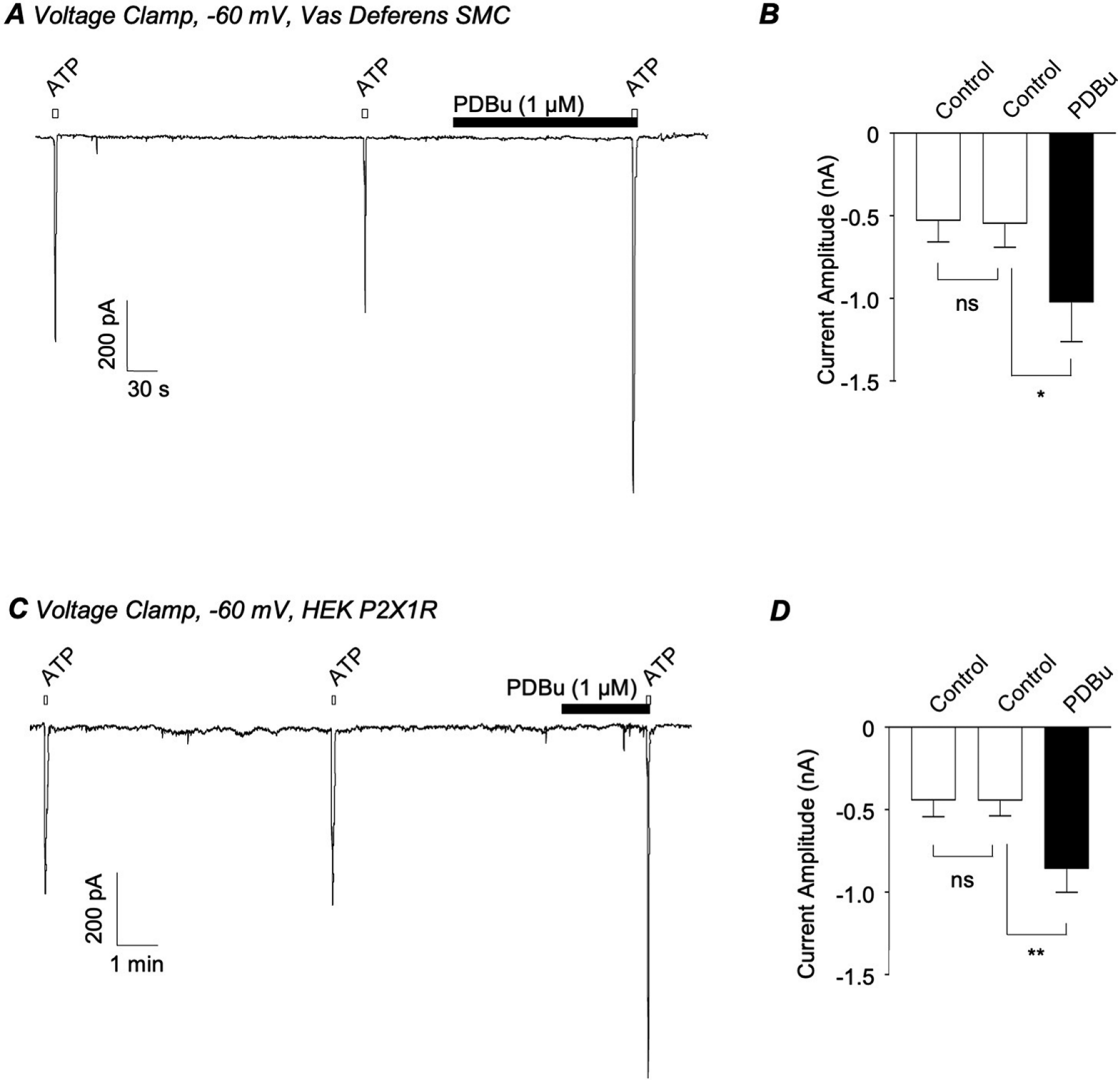
*A*, representative recording from an isolated vas deferens smooth muscle cell voltage-clamped at -60 mV showing currents evoked by ATP (1 µM) before and during the presence of PDBu (1 µM). *B*, summary bar chart plotting mean amplitude of ATP-evoked currents under control conditions and in the presence of PDBu. *C*, representative recording from a HEK-293 cell expressing human P2X1Rs voltage-clamped at -60 mV showing currents evoked by ATP (1 µM) before and during the presence of PDBu (1 µM). *D*, summary bar chart plotting mean amplitude of ATP-evoked currents under control conditions and in the presence of PDBu. Error bars represent SEM. *p < 0.05; **p < 0.01

ATP-induced contractions of the vas deferens are mediated by activation of postjunctional P2X1Rs, but it is not known if these channels can be modulated by PKC in VDSMC. Figure 6A shows the effect of PDBu (1 µM) on ATP-evoked currents recorded from a freshly isolated VDSMC held at -60 mV. Application of ATP (1 µM) for 5 s, at 4-minute intervals, evoked reproducible inward currents. When ATP was reapplied in the presence of PDBu the amplitude of the ATP-induced inward current nearly doubled. In 6 cells, mean current amplitude increased by 93% from − 531 ± 127 pA under control conditions to -1024 ± 238 pA in the presence of PDBu (*p* < 0.05; *N* = 5; *n* = 6, Fig. 6B). PDBu also increased the amplitude of ATP-induced currents recorded from HEK-293 cells over-expressing hP2X1Rs (Fig. 6C). In 6 experiments, mean P2X1R current amplitude was − 445 ± 93 pA under control conditions compared to -858 ± 141 pA in the presence of PDBu (*p* < 0.01; *n* = 6; Fig. 6D).

## Discussion

The results of the present study demonstrate that activation of α_1_-ARs exerts a powerful stimulatory effect on purinergic nerve-evoked ‘twitch’ contractions of the mouse vas deferens. Prazosin reduced the amplitude of transient EFS-evoked contractions by 69%, contrasting with previous studies of guinea-pig and rat vas deferens which found that the transient component of EFS-evoked contractions were unaffected by blockade of α_1_-ARs [9–12] or on rat vas deferens which reported a 14% reduction in the presence of prazosin [13]. The primary focus of our study was to investigate the mechanisms underlying the stimulatory effect of α_1_-AR activation on the purinergic responses.

Smith and Burnstock, (2004) showed that the synergistic effects of noradrenaline on ATP-induced contractions of the guinea-pig vas deferens were mimicked by PDBu, a PKC activator and were reduced by calphostin C, a PKC inhibitor, suggesting an important role for PKC activation in these effects [14]. Similar results were reported by Khattab et al., (2007) using chelerythrine, a different PKC inhibitor [24]. In contrast, Fujita et al., (1995) found that although PDBu and noradrenaline both induced Ca^2+^ sensitisation of β-escin-permeabilised guinea-pig vas deferens, the effects of noradrenaline were not reduced by a PKC inhibitor peptide [15]. We investigated if PKC was involved in the potentiation of ATP responses in the present study using the selective PKC inhibitor GF109203X. We found that ATP-evoked contractions of mouse vas deferens were enhanced by PDBu and that these effects were reduced by GF109203X. These data confirmed that activation of PKC could enhance purinergic contractions of the vas deferens and that GF109203X (1 µM) was effective at inhibiting the effects of PKC activation. However, inhibition of PKC with GF109203X only reduced the stimulatory effects of PE on ATP responses by 40%. While this difference may reflect incomplete inhibition of PKC induced by PE, it is also possible that the stimulatory effects of PE involves other pathways, in addition to activation of PKC.

α_1_-AR are coupled to Gq-proteins, the activation of which leads to production of inositol 1,4,5-trisphosphate (IP_3_) and diacylglycerol (DAG) via hydrolysis of phosphatidylinositol 4,5-bisphosphate (PIP_2_) by phospholipase C (PLC). IP_3_ and DAG induce smooth muscle contraction via multiple signalling pathways, however there is now increasing recognition that PIP_2_ can act as an unmodified ligand for Gq-protein-coupled receptor signalling in vascular smooth muscle cells [25] and it can influence smooth muscle excitability by regulation of ion channels that affect membrane potential [26–29]. For example, PIP_2_ is required for opening of Kv7 channels [30, 31] and agents that deplete PIP_2_ lead to increased excitability of nerves via closure of Kv7 channels [32–34]. The role of PIP_2_ in regulation of contraction of the vas deferens is currently unknown, however it is recognised that purinergic nerve-mediated contractions of the vas deferens rely on Ca^2+^ influx via voltage-dependent Ca^2+^ channels [35]. Therefore, it is conceivable that the stimulatory effects of α_1_-AR activation on purinergic responses in the present study could involve depletion of PIP_2_ leading to activation of pathways, such as inhibition of Kv7 channels, that promote Ca^2+^ influx via VDCC. However, further work would be required to test this idea and therefore it must remain speculative at present.

Smith & Burnstock, (2004) proposed that stimulation of PKC enhanced ATP-induced contractions of the guinea-pig vas deferens by sensitising smooth muscle cells to Ca^2+^ via inhibition of myosin light chain phosphatase (MLCP) [14]. The results of our study show that activation of PKC augmented P2X1 currents in vas deferens myocytes and, while findings in isolated cells may not necessarily translate to the whole tissue level, it is possible that the enhanced contractile responses to ATP induced by phenylephrine and PDBu involve potentiation of P2X1 currents. P2X1Rs possess a conserved PKC binding site [36] and Vial et al., (2004) showed that activation of PKC with the phorbol ester PMA, potentiated P2X1R-mediated currents in HEK-293 cells approximately two-fold [37]. Interestingly however, these effects were not prevented by mutation of the PKC binding site and it was instead suggested that the effects may involve phosphorylation of an accessory protein that regulates P2X1R function. It is possible that a similar mechanism may underlie the effects of PDBu in the present study, however the identity of such a protein remains to be elucidated.

In summary, our data suggest that activation of α_1_-ARs augments purinergic nerve-evoked contractions of the mouse vas deferens by enhancement of P2X1 currents by PKC and other pathways not involving PKC.

## Electronic supplementary material

Below is the link to the electronic supplementary material.

Supplementary Material 1

## Data Availability

The data that support the findings of this study are available from the corresponding author upon reasonable request.
