# Supplementary figures and images for: Regulation of nerve-evoked contractions of the murine vas deferens
Source: Purinergic Signal. 2024 Feb 20;20(5):547–57. doi: 10.1007/s11302-024-09993-y (PMC11377391; doi:10.1007/s11302-024-09993-y)

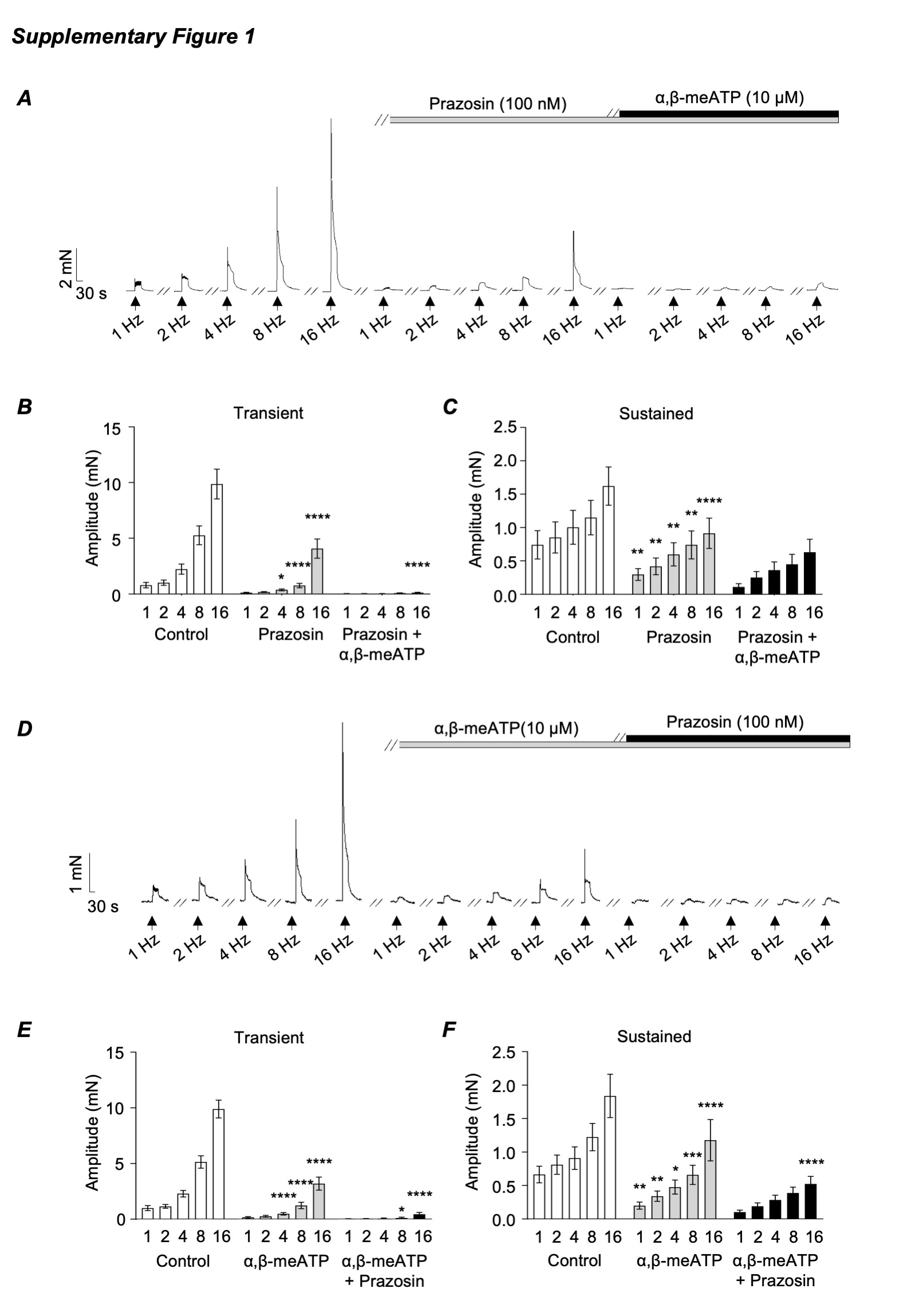

Supplement: Supplementary file 1 — Supplementary Material 1 [file 11302_2024_9993_MOESM1_ESM.jpeg]
